# Psoriasis

**Published:** 1903-04-11

**Authors:** 


### PSORIASIS.

The rational management of any complaint must of necessity involve some workable theory of the nature of the causative agent. In those illnesses where such a factor is known, the treatment is usually directed along certain definite and well recognised lines; in other cases the treatment adopted must be more or less experimental, and it is well therefore to know what amount of success or otherwise has been attained by the various remedial measures employed. This is especially the case with psoriasis among the skin diseases. In dealing with this affection Dr. Freeman [1] insists on the value of rest as a therapeutic measure. There are few cases, he says, that will not yield to a six weeks' course of lying in bed, even where remedies, both external and internal, are withheld. It is reasonable to infer that the improvement is due to the soothing of part or the whole of the nervous system, and one cannot help thinking of the vasomotor centre. Some of the worst cases he has had have been in neurotic patients, and it is especially these who are liable to suffer from such a complication as an exfoliative dermatitis. The internal remedies which are in common use are salicin, sodium salicylate, aspirin, thyroid extract, arsenic, potassium, iodide and ichthyol. The external are chrysarobin, some form of tar, salicylic acid, pyrogallic acid, sulphur, carbolic acid, ichthyol and mercury. Treatment by $x$-rays is being experimented with; however Dr. Freeman does not expect much benefit from this form of treatment. In all acute cases, and particularly those of the inflammatory type, rest in bed should be enforced until the disease has nearly disappeared; this should be combined with a daily half-hour's soaking in a hot bath containing 2 drachms of borax or carbonate of soda and 1 oz. of starch to each gallon of water. If the disease be of the seborrhœic type then 1 drachm to 2 drachms of sulphurated potash should be added, but this addition should be carefully made and the results watched. Arsenic should be avoided, but salicin is useful given in 10-grain doses to start with, and increasing up to 40 grains. To neurotic patients 5 grains each of the bromides of ammonium, sodium and potassium should be given three times a day for several days. The diet should be light, nourishing and unstimulating. Such external remedies as chrysarobin, tar, and salicylic acid are strongly contra-indicated in acute cases of the inflammatory type. Where the psoriasis is chronic, dry, and of the scaly variety occurring in a young and robust patient thyroid extract is valuable; it should be combined with moderate doses of arsenic, as it is then better borne. Thyroid extract should not be given to a person past middle age for the treatment of psoriasis. In weekly patients, young or old, arsenic is the best internal remedy, and should be combined with bromides, if there is any neurotic tendency. Alkaline baths would be used at the same time in order to remove the scales, and chrysarobin or some other external remedy rubbed in night and morning. Chrysarobin should not be applied to the face or neck. Of tar preparations probably the liquor carbonis detergens is as good as any. It is impossible to be dogmatic about the treatment; every case must be treated on its merits. With regard to prognosis, it should always be borne in mind that the disease, even if apparently cured for the time being, is exceedingly likely to relapse, and the patient should be advised accordingly.

[1] Edin. Med. Jour., April.

